# Activity of Cysteamine against the Cystic Fibrosis Pathogen Burkholderia cepacia Complex

**DOI:** 10.1128/AAC.01198-16

**Published:** 2016-09-23

**Authors:** Douglas Fraser-Pitt, Derry Mercer, Emma Lovie, Jennifer Robertson, Deborah O'Neil

**Affiliations:** NovaBiotics Ltd., Craibstone, Bucksburn, Aberdeen, United Kingdom

## Abstract

There are no wholly successful chemotherapeutic strategies against Burkholderia cepacia complex (BCC) colonization in cystic fibrosis (CF). We assessed the impact of cysteamine (Lynovex) in combination with standard-of-care CF antibiotics *in vitro* against BCC CF isolates by the concentration at which 100% of bacteria were killed (MIC_100_) and checkerboard assays under CLSI standard conditions. Cysteamine facilitated the aminoglycoside-, fluoroquinolone- and folate pathway inhibitor-mediated killing of BCC organisms that were otherwise resistant or intermediately sensitive to these antibiotic classes. Slow-growing BCC strains are often recalcitrant to treatment and form biofilms. In assessing the impact of cysteamine on biofilms, we demonstrated inhibition of BCC biofilm formation at sub-MIC_100_s of cysteamine.

## INTRODUCTION

Colonization of the airways with Burkholderia cepacia complex (BCC) is a major contributory factor to patient morbidity and mortality in cystic fibrosis (CF) (1–3), reducing life expectancy of those affected (4% of the adult CF population) by as much as 16 years (2, 4). Most, if not all, BCC strains (5) are clinically resistant or inherently insensitive to currently available CF antibiotics (2, 6, 7). BCC colonization is a significant clinical challenge in CF that will increase as survival rates for this condition continue to improve (8, 9). There remains a critical, unmet need for new chemotherapeutic approaches to resolving BCC colonization in CF.

We previously described the antimicrobial, antibiofilm, and mucolytic attributes of cysteamine against Pseudomonas aeruginosa and other CF bacterial pathogens (10–12) and report here that, more strikingly, the modification of currently available therapeutic strategies (2, 13) with the introduction of cysteamine as an adjunct antimicrobial agent brings about effective killing of BCC (both type strains and CF isolates).

## MATERIALS AND METHODS

### Bacterial strains and reagents.

The 36 Burkholderia strains employed for this study include representative strains from each species (or genomovar), including B. cenocepacia and B. multivorans, those most commonly associated with infection in cystic fibrosis (14). Sixteen of the BCC clinical isolates were sourced from Aberdeen Royal Infirmary, with seven from adult patients and nine pediatric isolates. Eight strains were sourced from the Glasgow adult patient cohort. An additional 12 type strains were included in this study. Cysteamine, trimethoprim, sulfamethoxazole, and ciprofloxacin were sourced from Sigma-Aldrich (United Kingdom), and tobramycin and ceftazidime were sourced from Discovery Fine Chemicals (United Kingdom). All other reagents were purchased from Sigma-Aldrich (United Kingdom).

### MIC and checkerboard assays.

The concentration at which 100% of bacteria were killed (MIC_100_) for all BCC isolates was determined for cysteamine and the antibiotics tobramycin, ciprofloxacin, ceftazidime, and trimethoprim-sulfamethoxazole using the CLSI broth microdilution procedure (15). Checkerboard assays of cysteamine and antibiotics were conducted according to the method of Burkhart et al. (16). Antibiotic susceptibility profiling of BCC (resistant, intermediate, or sensitive to antibiotics) was performed using CLSI performance standards for antimicrobial susceptibility testing using interpretive standards for other non-Enterobacteriaceae (17).

### Biofilm assays.

The crystal violet method for detecting the adherence of bacterial biomass to polypropylene 96-well plates was adapted from similar studies (18–20). Inocula were prepared using a McFarland standard equivalent of 5 × 10^5^ CFU/ml from growing cultures (according to CLSI document M07-A9 [15]) into 100 μl of cation-adjusted Mueller-Hinton broth containing appropriate concentrations of test antibiotic. Cultures were incubated statically for 48 h in a humidified atmosphere at 37°C to establish biofilms. Culture medium containing planktonic bacteria was carefully removed and discarded, and the plates were washed gently three times with 150 μl of sterile phosphate-buffered saline (PBS) prior to air drying for 1 h. The remaining attached bacteria in wells were then stained with 200 μl of 1% crystal violet solution for 2 min, prior to 3 further washes with PBS and solubilization with 200 μl of ethanol. Plates were then read at 595 nm.

Assessment of cysteamine-mediated biofilm prevention in a shear-flow environment was performed using a BioFlux 200 automated microfluidic system (Fluxion Biosciences, CA). Inocula were prepared as described above (CLSI document M07-A9 [15]) from growing cultures and seeded into prewarmed cation-adjusted Mueller-Hinton broth with or without the sub-MIC_100_ concentration of 128 μg/ml of cysteamine. A 48-well Bioflux plate was primed for 1 min with prewarmed cation-adjusted Mueller-Hinton broth prior to addition of treated and untreated cultures. Medium was passed through capillaries at 37 μl/h (0.4 dyne) for 20 h, and images were captured using an Axiovert 40CFL microscope (Carl Zeiss, United Kingdom) and camera and Bioflux 200 software.

## RESULTS

MIC assays with combinations of cysteamine and the recommended CF antibiotics were performed to assess the utility of cysteamine *in vitro* as an adjunct antibiotic therapy against BCC. A panel of BCC clinical isolates and type strains was tested to determine genomovar-specific effects and any differences in cysteamine-mediated effects between type strains and clinical isolates associated with colonization in two major United Kingdom patient cohorts. The 36 strains tested were resistant *in vitro* to at least one antibiotic selected from tobramycin, ciprofloxacin, trimethoprim-sulfamethoxazole, and ceftazidime (25 out of 36). The majority of clinical strains of CF origin were found to be resistant to tobramycin; perhaps this is not surprising considering the status of tobramycin as a mainstay, long-term antibiotic intervention in CF. Cysteamine potentiated the activity of tobramycin against 33 of 36 BCC isolates and type strains tested and reversed resistance/insensitivity in 17 of those strains (Table 1), all 17 being CF isolates. Cysteamine also potentiated the activity of ciprofloxacin against 21 of 36 BCC isolates tested and reversed resistance/insensitivity in 10 strains that were not sensitive to ciprofloxacin (Table 2). Only two isolates (B. dolosa DSMZ 16088 and a clinical isolate of B. multivorans CFSYN 945) remained resistant to ciprofloxacin in the presence of cysteamine.

**TABLE 1:**
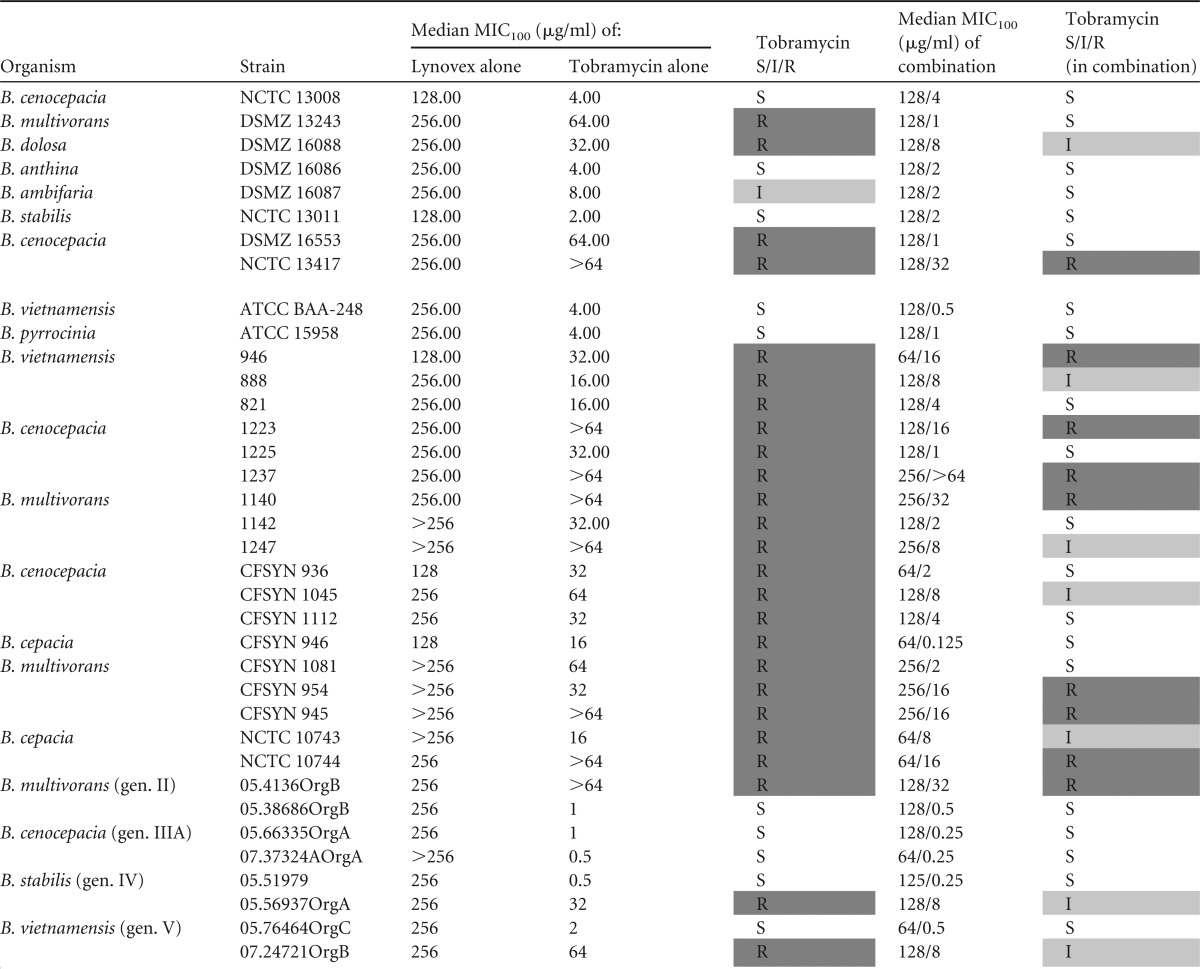
Antimicrobial activities of tobramycin and tobramycin in combination with cysteamine against Burkholderia isolates^*a*^

a All results represent the MIC_100_s from triplicate samples from triplicate experiments. Determination of susceptibility, intermediate status, or resistance (S/I/R) was based upon CLSI interpretive criteria. All data manipulation was carried out in Microsoft Excel. gen., genomovar. Strains with the resistance or intermediate status are highlighted by shading.

**TABLE 2:**
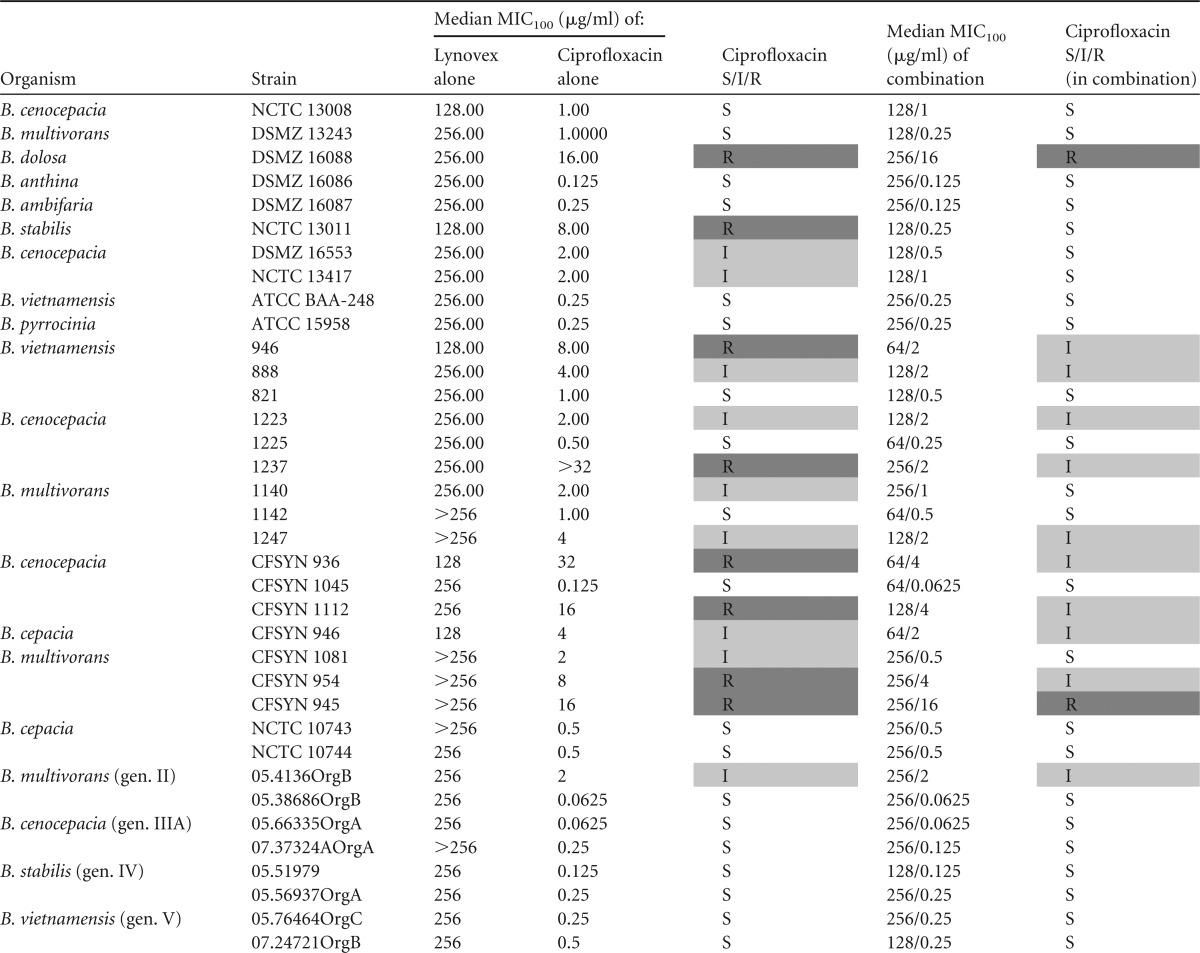
Antimicrobial activities of ciprofloxacin and ciprofloxacin in combination with cysteamine against Burkholderia isolates^*a*^

a All results represent the MIC_100_s from triplicate samples from triplicate experiments. S/I/R determination was based upon CLSI interpretive criteria. All data manipulation was carried out in Microsoft Excel. Resistant or intermediate results are highlighted with shading.

In addition to this impact on widely used antibiotics, the ability of cysteamine to enhance the activity of ceftazidime and trimethoprim-sulfamethoxazole, antibiotics used specifically to treat BCC, was also investigated (Tables 3 and 4). Interestingly, and pointing to an antibiotic class-specific effect, cysteamine had no major impact on ceftazidime susceptibility of the BCC strains tested in this system. In contrast, cysteamine potentiated the activity of trimethoprim-sulfamethoxazole against the majority (22 out of 36) of BCC isolates and type strains studied.

**TABLE 3:**
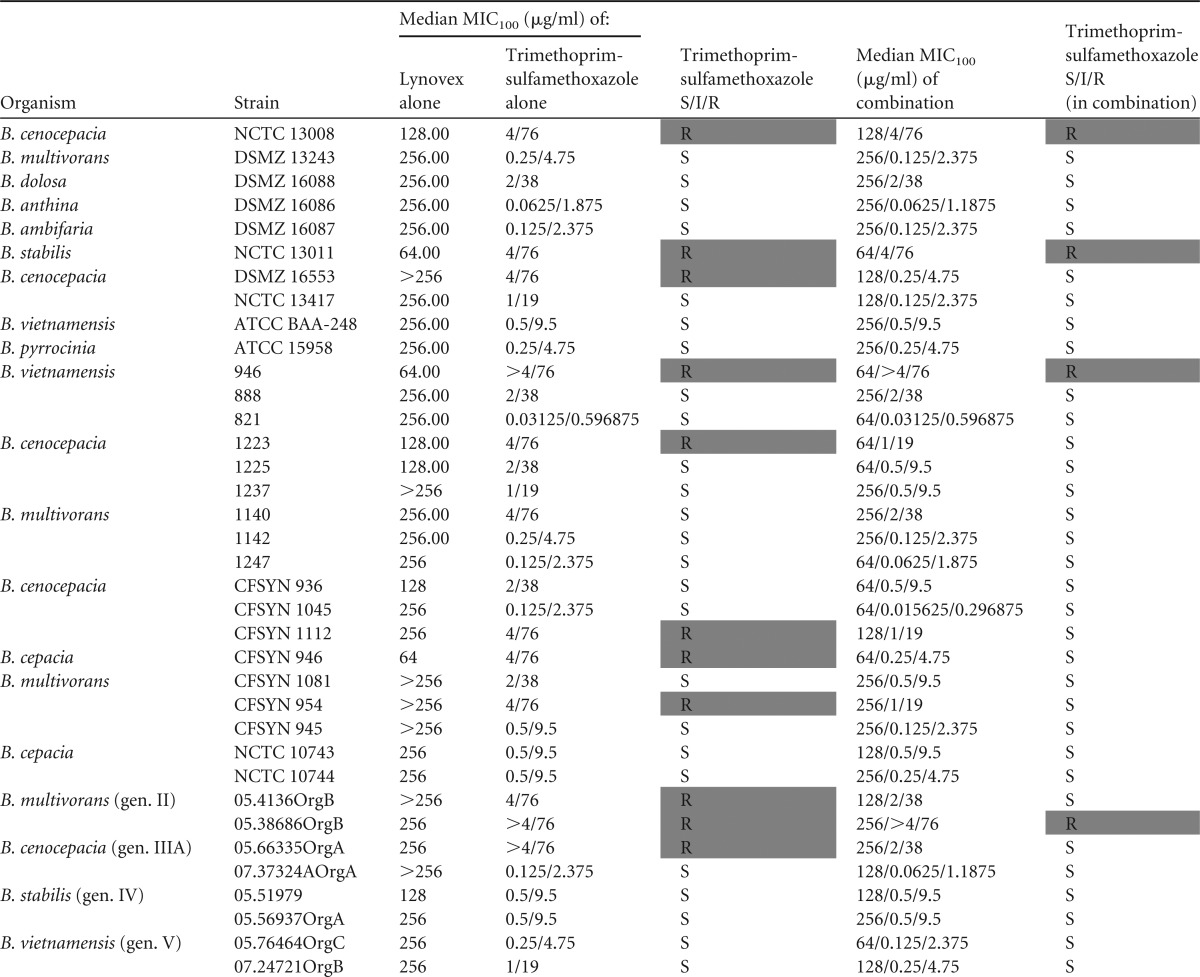
Antimicrobial activities of trimethoprim-sulfamethoxazole and trimethoprim-sulfamethoxazole in combination with cysteamine against Burkholderia isolates versus S/I/R determinations based upon CLSI interpretive criteria^*a*^

a All results represent the MIC_100_s from triplicate samples from triplicate experiments. All data manipulation was carried out in Microsoft Excel. Resistant or intermediate results are highlighted by shading.

**TABLE 4:**
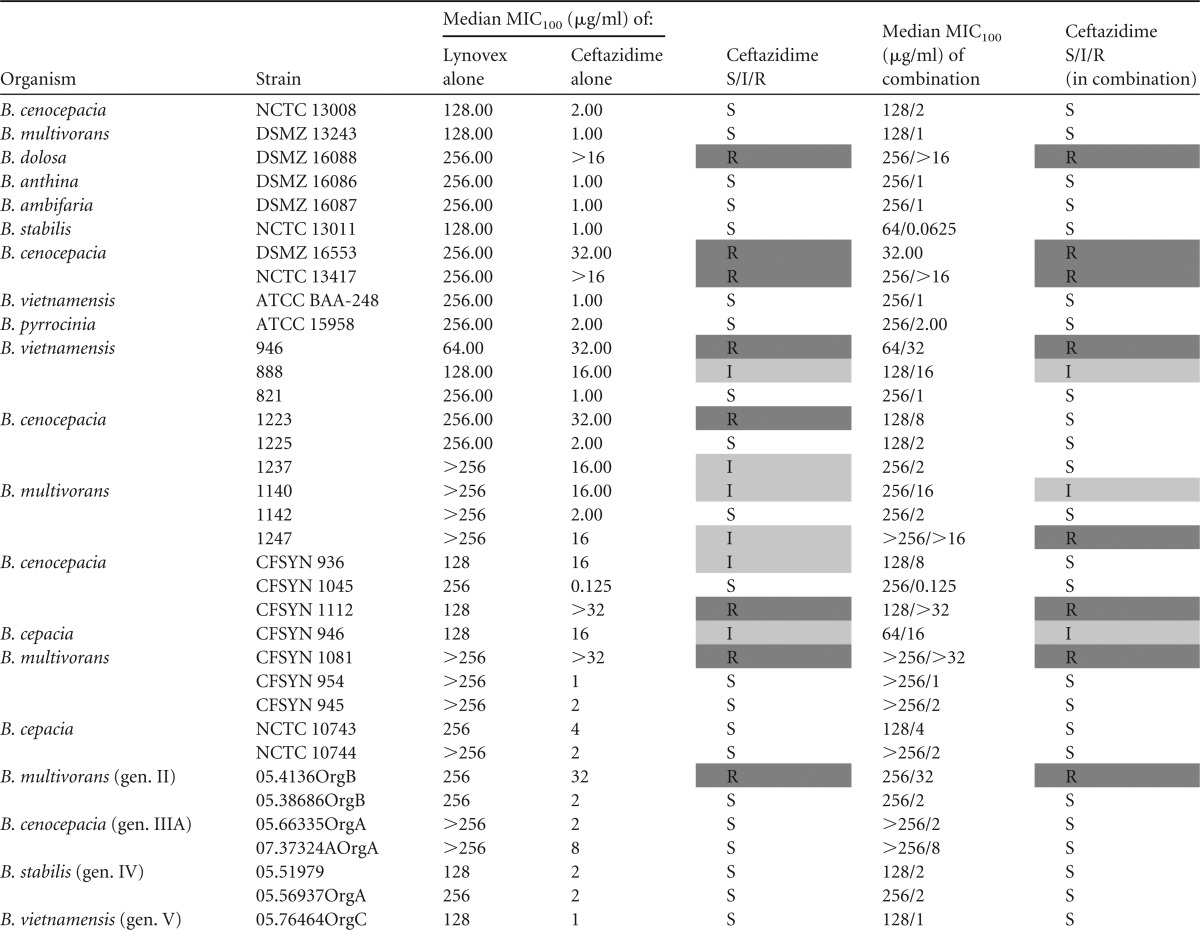
Antimicrobial activities of ceftazidime and ceftazidime in combination with cysteamine against Burkholderia isolates^*a*^

a All results represent the MIC_100_s from triplicate samples from triplicate experiments. S/I/R determination was based upon CLSI interpretive criteria. All data manipulation was carried out in Microsoft Excel. Strains with resistant or intermediate results are highlighted by shading.

**FIG 1:**
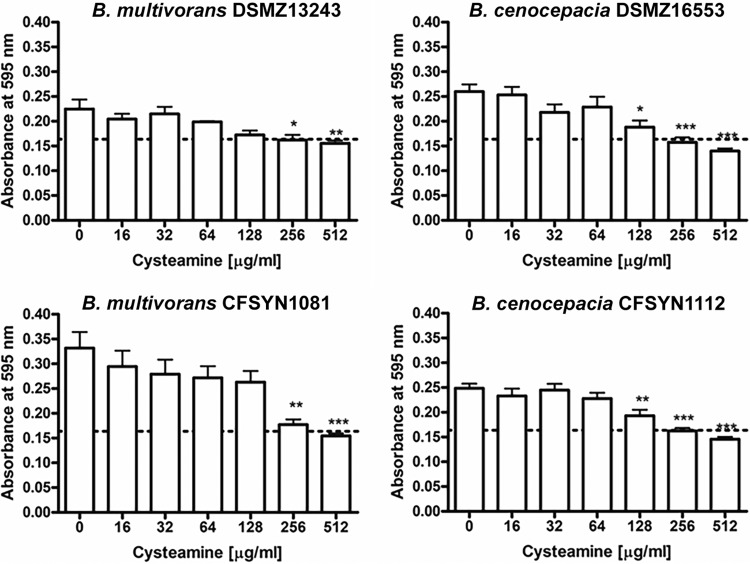
Cysteamine inhibits adherence of BCC strains at concentrations below the MIC_100_ as detected by crystal violet assay. One-way analysis of variance with Tukey's posttest analysis showed significant differences as follows: *, *P* < 0.05; **, *P* < 0.01; and ***, *P* < 0.001 (compared to untreated medium-only controls [*n =* 6]).

Planktonic cells were employed in our initial cysteamine adjunct assays, whereas *in vivo*, BCC colonizes the CF airway in biofilm form (21, 22). We have already described the antibiofilm properties of cysteamine against Pseudomonas (10–12). In order to confirm any activity of cysteamine specifically against BCC biofilm structures *in vitro*, we assessed its ability to prevent BCC biofilm formation using the Bioflux microfluidic system, and we used the crystal violet method for assessing biomass in 96-well microtiter plates. In both systems, the methodology was adapted to keep conditions as close as possible to CLSI standards for MIC testing rather than to favor the growth of biofilms. For example, the medium used was cation-adjusted Mueller-Hinton broth as opposed to other choices, such as tryptic soy broth, which would favor biofilm formation in BCC (23). This was to clearly demonstrate inhibition of attachment at sub-MIC_100_s. Crystal violet assessment demonstrated inhibition of bacterial attachment at 48 h in the presence of concentrations of cysteamine subinhibitory for planktonic growth (Fig. 1). Inhibition of biofilm formation was dose dependent, increasing with concentrations of cysteamine approaching the MIC, with significant inhibition of both strains of B. cenocepacia tested at 128 μg/ml. The antibiofilm activity of cysteamine against BCC was confirmed in real time in a dynamic-flow microfluidics system. The effect of a subinhibitory concentration of cysteamine was assessed on biofilm formation in the BioFlux microfluidic system on B. cenocepacia clinical strain CFSYN 1112. Cysteamine prevented biofilm formation in cells in channels that were exposed to cysteamine over 20 h compared to samples in wells not exposed to cysteamine (see Movie S1 in the supplemental material). Viable planktonic cells from the outlet well were cultured at the end of the experiment, confirming an effect on attachment and not bacterial viability at the test concentration.

## DISCUSSION

The findings of our current study point to the potential of cysteamine as a means to resolve or prevent BCC colonization through a simple and sustainable modification to the current standard of care in CF. Tobramycin and ciprofloxacin are mainstays of the CF antibiotic regimen, and resistance to these antibiotics in CF BCC strains is common and inherent in some strains (24) and is readily selected for (25); indeed, we demonstrate that all but one of the strains (NCTC 13008) tested in this study which had been isolated from a patient with CF were resistant to tobramycin treatment. Cysteamine was able to reverse the tobramycin and ciprofloxacin resistance phenotype and improve sensitivity to co-trimoxazole treatment (Tables 1 to 3). Therefore, cysteamine has the potential to extend the target spectrum of these antibiotics to include BCC. This is timely as regards its potential use as an adjunct with tobramycin considering the recent increased interest in the reapplication of inhaled or high-dose tobramycin against BCC (26, 27). Furthermore, the activity of antibiotics specifically deployed against BCC, such as trimethoprim-sulfamethoxazole, can also be further potentiated by cysteamine. Interestingly, ceftazidime activity was not altered by cotreatment with cysteamine, which suggests an antibiotic class-specific effect, at least within the *in vitro* systems employed in this study to assess antimicrobial activity.

The slow-growing, biofilm-forming characteristic of BCC contributes to the recalcitrance of this organism to existing antibiotic chemotherapy. In this study, we followed our previous work on the interactions of cysteamine with biofilm (10) in addition to assessing any direct antimicrobial activity. Cysteamine inhibited bacterial attachment at concentrations below the MIC_100_ for each strain tested, with significant inhibition of B. cenocepacia type strain DSMZ 16553 and clinical isolate CFSYN 1112. Interestingly, although we previously demonstrated that combinations of tobramycin and cysteamine were more effective in biofilm prevention and eradication for Pseudomonas aeruginosa, the addition of antibiotics did not enhance the antibiofilm activity of cysteamine against BCC (data not shown). Cysteamine was not able to disrupt existing biofilms in the slower-growing BCC strains over 48 h over the same range of concentrations of the antibiotics as tested in this *in vitro* system. This may indicate that cysteamine adjunct maintenance therapy may be better at preventing the establishment of BCC colonization in CF than at removing existing biofilms in chronically infected patients; however, the enhancement of antimicrobial activity may prove to be the more important feature of this compound in this situation. Further research to determine optimum antibiotic combinations and concentrations to eradicate established BCC biofilms may yet prove efficacious.

We purposely did not use an exhaustive panel of BCC strains for this study. We instead employed a focused set of clinically relevant CF isolates from two of the United Kingdom's specialist CF centers (8 isolates from Glasgow and 16 from Aberdeen) and an additional 12 type strains in order to cover all known BCC genomovars, regardless of clinical relevance.

Cysteamine is in late-stage clinical trials for the treatment of cystic fibrosis and is being developed in oral and inhaled forms for acute exacerbations and chronic longer-term maintenance (10–12). An oral form of cysteamine was investigated in an open-label clinical study (28) in the United Kingdom in which tolerability, absorption, pK, and early evidence of efficacy were assessed in adult CF patients with stable disease. A global two-part registration study for oral cysteamine in acute exacerbations is now being initiated (EudraCT no. 2015-0004986-99) for which endpoints will include the reduction in sputum microbial burden over and above that achieved with standard of care therapy (SOCT) exacerbation interventions.

Thus far, cysteamine appears to be a promising candidate treatment for CF, but how its interactions with all components of the complex CF microbiome contribute to its clinical effects is yet to be determined. We have already have demonstrated the utility of cysteamine against other, more common CF pathogens that are known to drive acute infectious exacerbations (Pseudomonas in particular) (29, 30). We believe that this study is important in confirming the efficacy of cysteamine against the more insidious BCC and its colonization of the CF airway, which may be eradicated and perhaps prevented by long-term use of an adjunct to SOCT such as cysteamine, which is able to potentiate the effects of existing antibiotics and “switch” BCC to becoming sensitive and also prevent this organism from forming biofilms. Not all BCC isolates tested in this study responded to cotreatment. As well as any strain-specific nuances in cysteamine response, the antibiotic class-specific differences in responses to cysteamine coexposure we have underpinned for BCC (and other organisms in our previous work) are the subject of further study.

## Supplementary Material

Supplemental material
