# Supplementary material for: Activity of Cysteamine against the Cystic Fibrosis Pathogen Burkholderia cepacia Complex
Source: Antimicrob Agents Chemother. 2016 Sep 23;60(10):6200–6. doi: 10.1128/AAC.01198-16 (PMC5038277; doi:10.1128/AAC.01198-16)
Supplement: Supplemental material [file supp_60_10_6200__index.html]

Activity of Cysteamine against the Cystic Fibrosis Pathogen Burkholderia cepacia Complex — Supplemental material 

# Activity of Cysteamine against the Cystic Fibrosis Pathogen Burkholderia cepacia Complex

## Supplemental material

- Supplemental file 1 -

  Movie S1: cysteamine prevents biofilm formation by *B. cenocepacia* CFSYN1112 at 128 µg/ml in Mueller-Hinton broth in the Bioflux microfluidic system when incubated at 37°C with a flow rate of 0.4 dyne over 24 h.

  MP4, 8.8M
